# Impact of COVID-19 pandemic on incidence of long-term conditions in Wales: a population data linkage study using primary and secondary care health records

**DOI:** 10.3399/BJGP.2022.0353

**Published:** 2023-03-07

**Authors:** Cathy Qi, Tim Osborne, Rowena Bailey, Alison Cooper, Joe P Hollinghurst, Ashley Akbari, Ruth Crowder, Holly Peters, Rebecca-Jane Law, Ruth Lewis, Deb Smith, Adrian Edwards, Ronan A Lyons

**Affiliations:** Population Data Science, Swansea University Medical School, Faculty of Medicine, Health & Life Science, Swansea University, Swansea.; Population Data Science, Swansea University Medical School, Faculty of Medicine, Health & Life Science, Swansea University, Swansea.; Population Data Science, Swansea University Medical School, Faculty of Medicine, Health & Life Science, Swansea University, Swansea.; Wales COVID-19 Evidence Centre, Division of Population Medicine, Cardiff University, Cardiff.; Population Data Science, Swansea University Medical School, Faculty of Medicine, Health & Life Science, Swansea University, Swansea.; Population Data Science, Swansea University Medical School, Faculty of Medicine, Health & Life Science, Swansea University, Swansea.; Directorate of Primary Care and Mental Health, Health and Social Services Group, Welsh Government, Cardiff.; Centre for Medical Education, Cardiff University, Cardiff.; Technical Advisory Cell, Health and Social Services Group, Welsh Government, Cardiff.; North Wales Centre for Primary Care Research, PRIME Centre Wales, Bangor University, Bangor.; Wales COVID-19 Evidence Centre, Division of Population Medicine, Cardiff University, Cardiff.; Wales COVID-19 Evidence Centre, Division of Population Medicine, Cardiff University, Cardiff.; Population Data Science, Swansea University Medical School, Faculty of Medicine, Health & Life Science, Swansea University, Swansea.

**Keywords:** anxiety, chronic disease, COVID-19, diagnosis, primary health care

## Abstract

**Background:**

The COVID-19 pandemic has directly and indirectly had an impact on health service provision owing to surges and sustained pressures on the system. The effects of these pressures on the management of long-term or chronic conditions are not fully understood.

**Aim:**

To explore the effects of COVID-19 on the recorded incidence of 17 long-term conditions.

**Design and setting:**

This was an observational retrospective population data linkage study on the population of Wales using primary and secondary care data within the Secure Anonymised Information Linkage (SAIL) Databank.

**Method:**

Monthly rates of new diagnosis between 2000 and 2021 are presented for each long-term condition. Incidence rates post-2020 were compared with expected rates predicted using time series modelling of pre-2020 trends. The proportion of annual incidence is presented by sociodemographic factors: age, sex, social deprivation, ethnicity, frailty, and learning disability.

**Results:**

A total of 5 476 012 diagnoses from 2 257 992 individuals are included. Incidence rates from 2020 to 2021 were lower than mean expected rates across all conditions. The largest relative deficit in incidence was in chronic obstructive pulmonary disease corresponding to 343 (95% confidence interval = 230 to 456) undiagnosed patients per 100 000 population, followed by depression, type 2 diabetes, hypertension, anxiety disorders, and asthma. A GP practice of 10 000 patients might have over 400 undiagnosed long-term conditions. No notable differences between sociodemographic profiles of post- and pre-2020 incidences were observed.

**Conclusion:**

There is a potential backlog of undiagnosed patients with multiple long-term conditions. Resources are required to tackle anticipated workload as part of COVID-19 recovery, particularly in primary care.

## INTRODUCTION

The COVID-19 pandemic has had both a direct and indirect impact on the health and care system.^1^ Direct effects are those of COVID-19-related illnesses.^2^ Indirect effects are highly heterogeneous and include delays in cancer services and postponement of elective surgery and other non-urgent treatments owing to surge pressures on the system. ^1^

For example, it has been estimated that around 28 million operations were cancelled or postponed globally during the peak 12 weeks of the pandemic’s first wave.^3^ The impact on non-urgent treatments include harm from cessation or delay of screening services and the management of long-term conditions.^1^

A ‘long-term’ or chronic condition is a condition that cannot presently be cured but is controlled by medication and/or other treatment/therapies, for example, diabetes and asthma.^4^ Long-term conditions are associated with increasing age and deprivation, and the number of people with multiple long-term conditions (multimorbidity) is increasing.^4^ Patients with long-term conditions are more intensive users of health and social care services, and before the pandemic accounted for 50% of GP appointments, 64% of outpatient appointments, and 70% of all inpatient bed days.^4^

In primary care, a call and recall system is used to manage long-term conditions, which is offered to patients after a specific diagnosis is made and recorded in condition registries. Primary care activity was substantially reduced in the early months of the pandemic and, when activity returned to more usual levels in late 2020, acute care displaced much planned care such as long-term condition monitoring and review.^5^ It is unknown whether this has resulted in ongoing delays in diagnosis and management for long-term conditions.

Routinely collected data provide an opportunity to examine changes in recorded diagnoses. The Secure Anonymised Information Linkage (SAIL) Databank (www.saildatabank.com) contains data from 84% of the GPs and all hospital inpatient and day case activity in Wales.^6^^–^^8^ In the current study, historic trends in the incidence rates of 17 long-term conditions were examined, and rates in 2020 and 2021 compared with expected rates over these 2 years had the previous trends continued without interruption. In addition, changes in the characteristics of patients with recorded diagnoses were examined to inform resource allocation.

**Table:** How this fits in

Studies have reported reduced recording of long-term or chronic condition incidence early in the COVID-19 pandemic. Evidence for the presence and the severity of lags in diagnoses across multiple long-term conditions during the pandemic, and the current status of these lags, is limited. Over 2020 and 2021, recorded incidence across multiple long-term conditions lagged behind projected expectations, representing a substantial backlog of undiagnosed patients, who are unlikely to be receiving systematic monitoring and management. Differences in the sociodemographic profile of diagnosed patients post-2020 compared with years pre-2020 were not evident, making targeted catch-up initiatives unlikely to be feasible.

## METHOD

This was an observational retrospective study reported according to the Strengthening the Reporting of Observational Studies in Epidemiology (STROBE) guidelines.

### Data sources

Anonymised individual-level, population-scale data sources were accessed within the SAIL Databank.^6^^–^^11^ Conditions treated in hospital are recorded using International Classification of Diseases version 10 (ICD-10) codes in the Patient Episode Dataset for Wales (PEDW) dataset. Diagnoses from GP records are coded using Read v2 codes in the Welsh Longitudinal General Practice (WLGP) dataset. The Welsh Demographic Service Dataset was used to link birth, death, sex, and lower layer super output area (LSOA). LSOAs are an output geography created for the 2011 Census and, on average, an LSOA contains the homes of 1500 residents.^12^ Ethnicity categories were identified from 26 linked data sources (Supplementary Table S1).

### Study cohort

Residents of Wales diagnosed for the first time with at least one of 17 long-term conditions between January 2000 and December 2021 were identified using ICD-10 or Read v2 codes (Supplementary Tables S2 and S3). The conditions included were anxiety disorders, asthma, atrial fibrillation, coronary heart disease (CHD), chronic kidney disease (CKD), chronic obstructive pulmonary disease (COPD), dementia, depression, diabetes mellitus, epilepsy, heart failure, hypertension, inflammatory bowel disease (IBD), osteoporosis, peripheral vascular disease (PVD), rheumatoid arthritis, and stroke and transient ischaemic attack (TIA). These conditions comprise most of the general practice ‘Quality and Outcomes (QOF) Framework’.^13^ In addition, individuals diagnosed with three diabetes subtypes (type 1, type 2, undetermined) were identified using an algorithm.^14^ ‘Undetermined type diabetes’ was assigned when criteria for type 1 or type 2 were not met.

The final study dataset excluded records missing week of birth or sex, or where the diagnosis date was before birth or after death dates.

### Variables

Monthly incidence was derived from the number of individuals diagnosed with a long-term condition for the first time, each month. Age at the earliest found diagnosis date was categorised (<20, 20–29, 30–39, 40–49, 50–59, 60–69, 70–79, 80–89, ≥90 years). Sex was male/female. Ethnic groups were analysed using harmonised Office for National Statistics (ONS) categories (White/Black/Asian/Mixed/other/unknown). Deprivation was derived from the LSOA code at the time of diagnosis mapped to the 2019 Welsh Index of Multiple Deprivation^15^ and categorised in quintiles (1, most deprived, to 5, least deprived).

Frailty was based on an internationally established cumulative deficit model that utilises an electronic Frailty Index *(*eFI).^16^^–^^18^ eFI scores were used to categorise individuals as: fit, mild, moderate, or severely frail using 10 years of previous WLGP data from date of diagnosis. Individuals without sufficient coverage of GP data were assigned to a missing category. Learning disability status (yes/no) was identified for the study cohort using Read v2 codes (Supplementary Table S4). Socioeconomic categories with one to four counts were rounded to five to prevent accidental disclosure and the excess counts deducted from an unknown/missing/adjacent category.

### Outcomes

The primary outcome measure was the monthly incidence rates for each long-term condition. This was derived for the full study period from January 2000 to December 2021. The primary analysis used data from January 2015 to December 2021; the primary outcome was the relative difference between observed and expected incidence rates from 2020 to 2021. The secondary outcome was the annual number and proportion of incident cases by each sociodemographic and clinical subgroup.

### Statistical analysis

Monthly incidence rates were derived from the number of new diagnoses occurring each month × 100 000/population size and presented descriptively for the full study period. Population size was estimated from individuals registered to GPs in Wales on 1 July of each year; a breakdown by age group, sex, and social deprivation was presented to check population stability over time. The population size of Wales published by the ONS^19^ was extracted to estimate coverage achieved by the GP-registered population size. Three-month rolling averages were derived from the mean rate of the month in question, the previous, and the following month. A seasonal autoregressive integrated moving average (SARIMA) model on monthly incidence data from January 2015 to December 2019 was fitted to predict the expected incidence rate (and 95% confidence intervals [CIs]) for each month in 2020 and 2021. Model selection is described in Supplementary Box S1. The difference between the total observed and predicted (lower and upper 95% CI bound) rates was calculated over the 2-year period, and for 2020 and 2021 separately. Percentage differences were (observed – expected) × 100/expected rates. Counts and percentages of individuals by demographic groups were presented for each year from 2000 to 2021, and for 2015–2019 and 2020–2021. Each of the 17 long-term conditions and three diabetes subgroups was examined and analysed separately. As sensitivity analyses, the primary analysis was repeated on the number of cases, unadjusted for population. Statistical analyses were performed using R V4.1.2.

### Public involvement

A public partner contributed public or patient perspective to stakeholder discussions at each stage of the study, including interpretation of the significance and potential impact of the results.

## RESULTS

**Figure 1.**
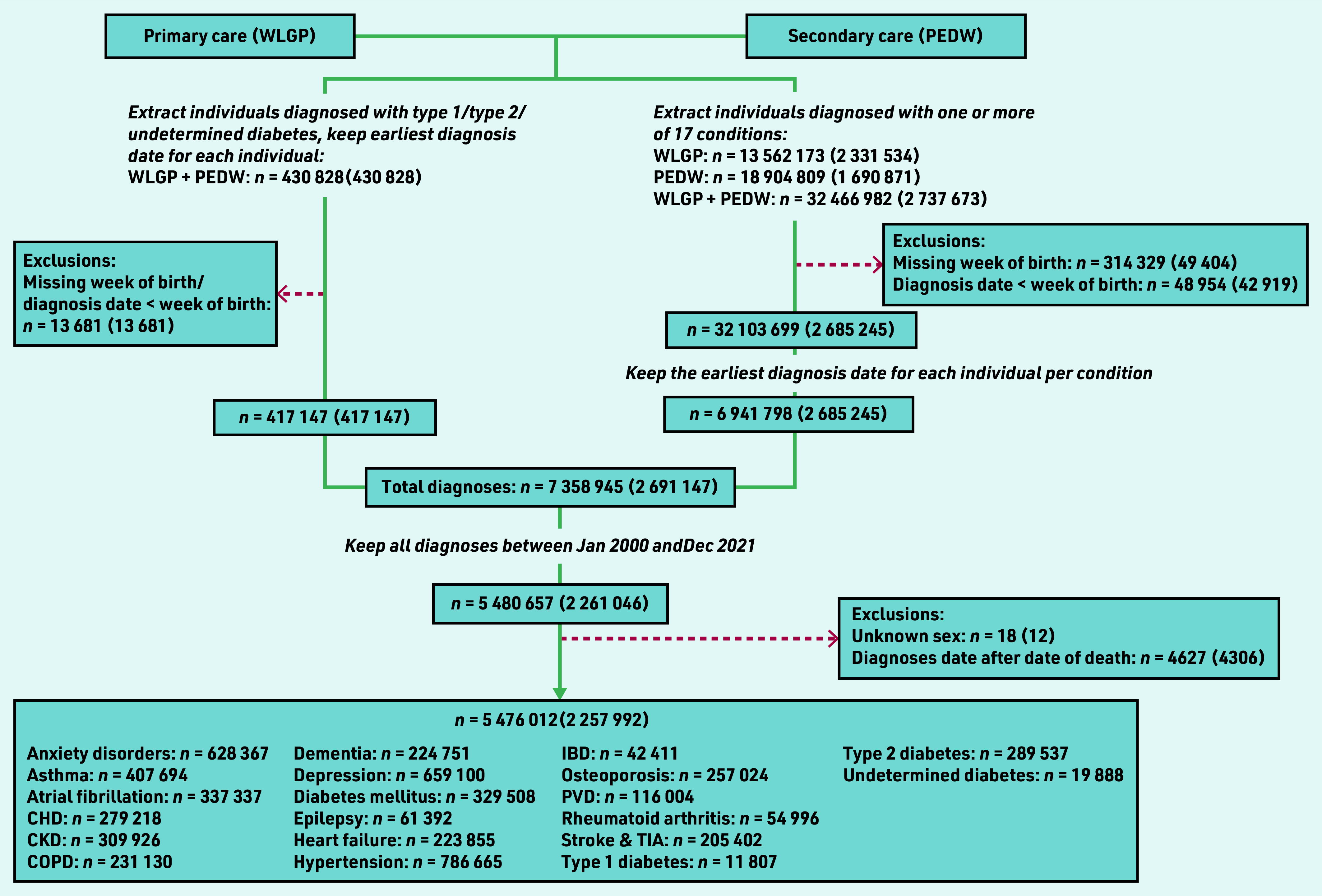
*Study flowchart: numbers presented are number of diagnoses (number of individuals). Data were extracted in two ways: (a) via using a ‘diabetes algorithm’ to identify individuals diagnosed with type 1, type 2, or undetermined type diabetes, (b) via using International Classification of Diseases (version 10) and Read codes to identify individuals diagnosed with one or more of 17 conditions (including diabetes mellitus). For (a), the identification algorithm selected the earliest diagnosis date per individual. For (b), the number of diagnoses refers to the number of unique diagnosis dates available, where a diagnosis date is defined as having one or more diagnosis codes recorded on that day. The final dataset included the earliest recorded diagnosis date for each individual per condition. CHD = coronary heart disease. CKD = chronic kidney disease. COPD = chronic obstructive pulmonary disease. IBD = inflammatory bowel disease. PEDW = Patient Episode Database for Wales. PVD = peripheral vascular disease. TIA = transient ischaemic attack. WLGP = Welsh Longitudinal General Practice.*

There were 5 476 012 diagnoses of long-term conditions identified between January 2000 and December 2021 belonging to 2 257 992 individuals after minor exclusions (Figure 1). Coverage of the population of Wales using GP data in SAIL (Supplementary Table S5) was high (>80% from 2003, and >85% from 2015). Supplementary Table S6 shows that population demographics in the GP population were generally stable from 2000 to 2021.

**Figure 2.**
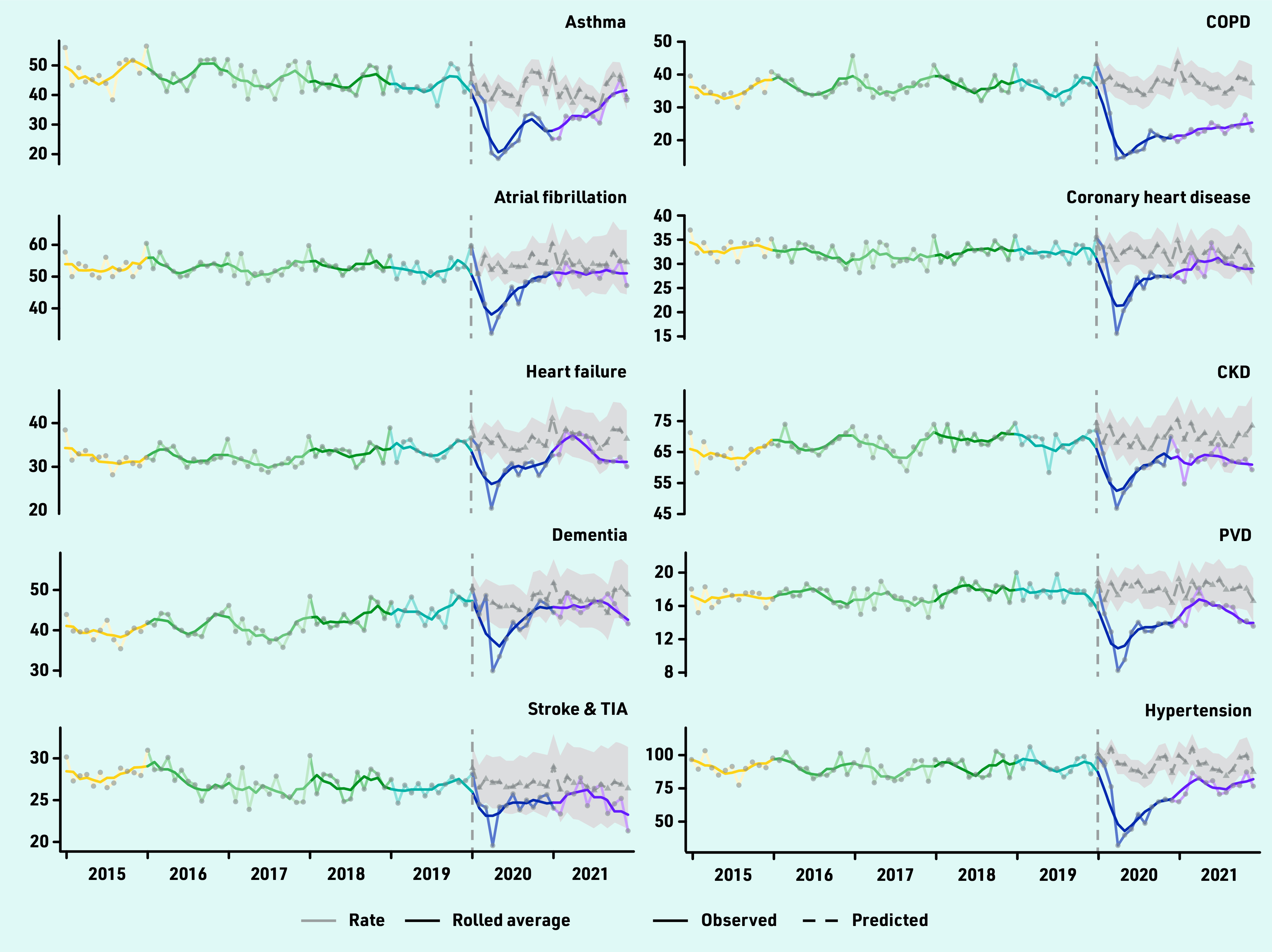

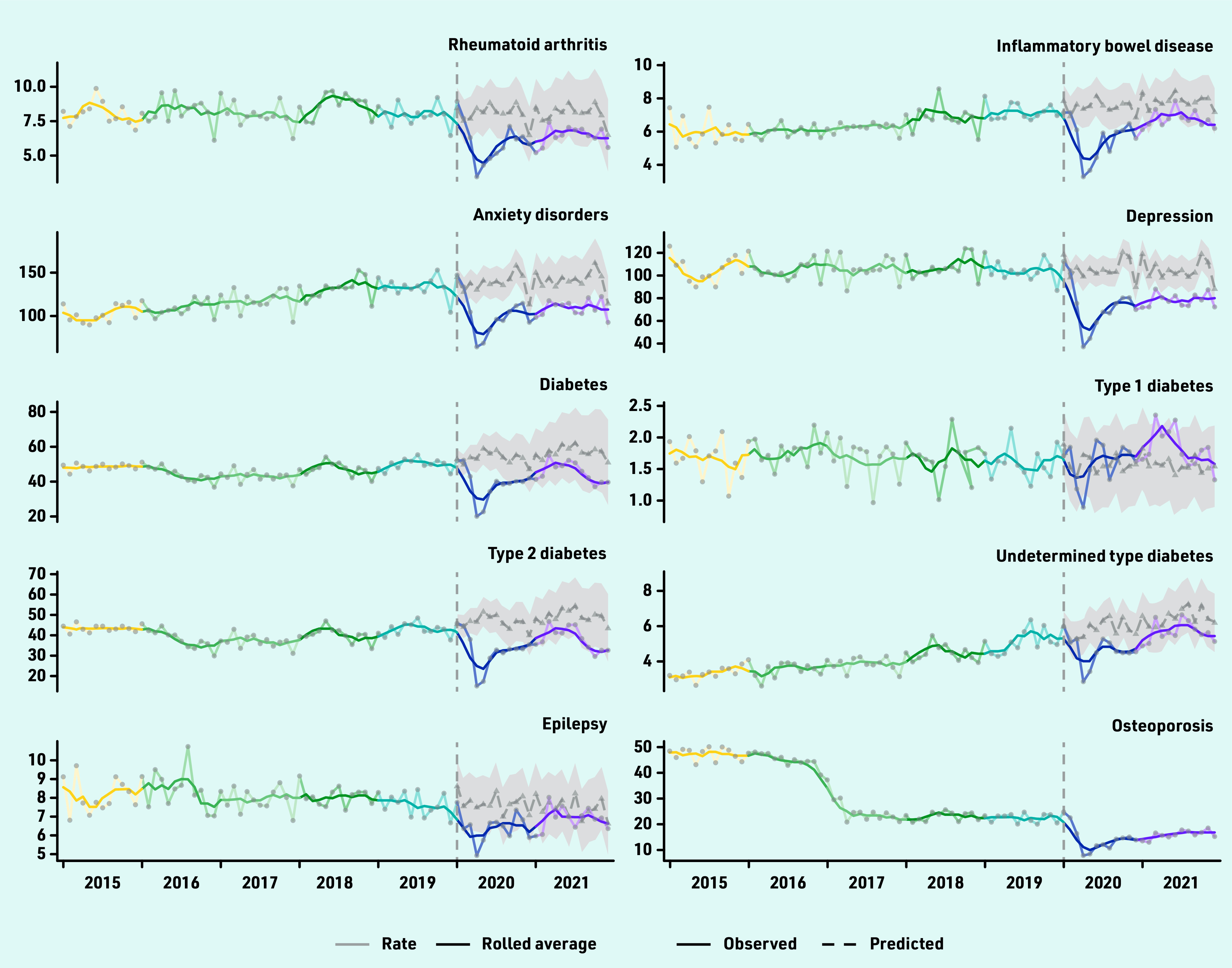
*Monthly observed number of diagnoses per 100 000 population from 2015 to 2021 for 17 long-term conditions and three diabetes subtypes (type 1/type 2/undetermined). For 2020 and 2021, monthly predicted number of diagnoses per 100 000 are also shown with 95% confidence intervals indicated by the shaded region. Monthly observed data are overlaid with 3-month rolling averages (solid line). CKD = chronic kidney disease. COPD = chronic obstructive pulmonary disease. PVD = peripheral vascular disease. TIA = transient ischaemic attack.*

A fully interactive dashboard showing incidence counts and rates from 2000 to 2021 for all 17 long-term conditions and diabetes subtypes is available here: https://envhe.shinyapps.io/wales-cec-ltc-incidence/ (source code: https://gitlab.com/envhe/wales-cec-ltc-incidence-shiny-dashboard). Figure 2 shows monthly incidence rates from 2015 to 2021, and predicted rates from 2020 by condition. There was an abrupt reduction around March to April 2020 across all conditions, followed by a general upward trend in subsequent months.

Table 1 shows the difference in the total observed and expected incidence rates over 2020–2021 by condition. Observed incidence was lower than mean expected incidence for all conditions, except type 1 diabetes. Predicted rates are not available for osteoporosis as a SARIMA model was not fitted because of inconsistent trends in 2015–2019 data.

**Table 1.** Total observed and predicted incidence rate per 100 000 population in 2020 and 2021 ^a^

**Condition**	**2020 and 2021**			
	**Observed**	**Predicted (95% CI)**	**Change (95% CI)**	**Percentage change (95% CI)**
**COPD**	549	892 (779 to 1005)	−343 (−456 to −230)	−38.4 (−45.4 to −29.5)
**Depression**	1800	2512 (2194 to 2830)	−712 (−1031 to −394)	−28.3 (−36.4 to −17.9)
**Type 2 diabetes**	837	1136 (871 to 1401)	−300 (−565 to −34)	−26.4 (−40.3 to −3.9)
**Hypertension**	1663	2231 (1979 to 2483)	−568 (−820 to −316)	−25.5 (−33 to −16.0)
**Anxiety disorders**	2503	3333 (2784 to 3882)	−830 (−1379 to −281)	−24.9 (−35.5 to −10.1)
**Asthma**	756	1006 (898 to 1114)	−250 (−358 to −142)	−24.9 (−32.2 to −15.9)
**Diabetes mellitus**	999	1314 (952 to 1676)	−315 (−677 to 47)	−24 (−40.4 to 4.9)
**Rheumatoid arthritis**	148	192 (142 to 243)	−45 (−95 to 6)	−23.1 (−39.0 to 4.0)
**PVD**	341	430 (375 to 485)	−90 (−145 to −35)	−20.8 (−29.8 to −9.2)
**Inflammatory bowel disease**	147	183 (152 to 214)	−36 (−67 to −5)	−19.8 (−31.4 to −3.4)
**Undetermined type diabetes**	123	147 (116 to 178)	−24 (−55 to 7)	−16.3 (−31.0 to 6.1)
**CHD**	671	774 (680 to 869)	−103 (−198 to −9)	−13.3 (−22.8 to −1.3)
**Heart failure**	756	871 (753 to 990)	−116 (−234 to 3)	−13.3 (−23.6 to 0.4)
**CKD**	1462	1678 (1496 to 1861)	−217 (−399 to −34)	−12.9 (−21.5 to −2.3)
**Epilepsy**	159	182 (143 to 220)	−23 (−61 to 16)	−12.4 (−27.9 to 11.4)
**Atrial fibrillation**	1158	1304 (1145 to 1463)	−146 (−305 to 13)	−11.2 (−20.8 to 1.1)
**Stroke and TIA**	592	647 (554 to 740)	−55 (−148 to 38)	−8.5 (−20.0 to 6.9)
**Dementia**	1050	1135 (991 to 1279)	−85 (−229 to 59)	−7.5 (−17.9 to 6.0)
**Type 1 diabetes**	41	38 (22 to 53)	3 (−12 to 19)	8.6 (−22.8 to 83.3)

a *Conditions are ordered from largest to smallest relative (%) change between observed and predicted rates. CHD = coronary heart disease. CI = confidence interval. CKD = chronic kidney disease. COPD = chronic obstructive pulmonary disease. PVD = peripheral vascular disease. TIA = transient ischaemic attack.*

Conditions with the largest relative deficit in diagnoses were COPD, depression, type 2 diabetes, hypertension, anxiety disorders, and asthma. Observed rates for COPD were 38.4% (95% CI = 29.5 to 45.4) lower than expected, corresponding to an undiagnosed population of 343 (95% CI = 230 to 456) per 100 000 individuals. Anxiety disorders had the largest absolute undiagnosed population of 830 (95% CI = 281 to 1379) per 100 000. Compared with 2020, estimated differences for 2021 were similar for COPD and anxiety disorders, and smaller, but with larger 95% CIs, among most other conditions (Supplementary Table S7). Figure 2 suggests that there may still be an overall lag in diagnoses in 2021 for most conditions. Incidence rates for some conditions were close to pre-pandemic levels by the end of 2021; others (for example, PVD and stroke and TIA) were approaching predicted rates near the start of 2021 but dropped again towards the end of the year.

The estimated rate of underdiagnosis for diabetes mellitus was 178 (95% CI = 57 to 299) in 2020 and 137 (95% CI = −104 to 378) in 2021, similar to corresponding estimates for type 2 diabetes of 168 (95% CI = 72 to 263) in 2020 and 132 (95% CI = −38 to 302) in 2021, whereas the estimated underdiagnosis for type 1 diabetes was 0 (95% CI = −8 to 7) in 2020 and −3 (95% CI = −11 to 5) in 2021.

Results from analysis of incidence counts unadjusted for population size (Supplementary Tables S8 and S9) were consistent with primary findings. SARIMA model specification and estimated parameters for analysis of incidence rates and counts are shown in Supplementary Tables S10 and S11, respectively.

Supplementary Tables S12 to S31 show annual incidence by sociodemographic factors from 2015 to 2021. The study dashboard (link before) includes data from 2000. There was no notable difference between the distribution of cases among categories in 2020 and/or 2021 compared with preceding years for any of the sociodemographic factors, indicating that, although overall rates of diagnosis decreased, influences of sociodemographic characteristics on being diagnosed did not drastically differ pre- and post-2020.

Type 1 diabetes was the only condition with an estimated mean net gain in incidence of 8.6% (95% CI = −22.8 to 83.3) (Table 1). Given that type 1 diabetes is diagnosed in younger patients (around 75% <50 years old), whether diagnosis trends differed between younger (<50 years) and older (>50 years) populations was investigated (Supplementary Figure S1).

Most conditions were rare in those aged <50 years (monthly rate <10 per 100 000), but among the remaining conditions, trends within age groups were similar to aggregate trends, including for depression, anxiety, and asthma. As further post hoc exploration, Supplementary Figures S2 and S3 show that incidence trends by sex and social deprivation groups were also similar.

## DISCUSSION

### Summary

From 2020 to 2021, there were deficits in recorded incidences across multiple long-term conditions, likely an indirect effect of the COVID-19 pandemic. Increasing demand and workforce vacancies could have affected availability of appointments and postponed diagnostic tests. A typical general practice of 10 000 patients might have over 400 undiagnosed long-term conditions (some potentially occurring in the same individuals). Observed incidence for some conditions (for example, heart failure and stroke and TIA) increased and declined again during 2021; this could reflect changes in healthcare pressures between the alpha wave (September 2020 to March 2021) and the delta wave (June 2021 to December 2021) in Wales. Other conditions were approaching pre-pandemic levels towards the end of 2021 (for example, asthma), which could reflect condition-specific ‘catch-up’ activity but an excess would be needed to reach net expected numbers.

### Strengths and limitations

This study included multiple conditions, mostly selected from the QOF framework, previously used to monitor and reward performance in primary care, thus electronic coding quality is generally good, although this can vary between individual clinicians and practices. Overall data coverage was close to the full population of Wales. The assumption that trends in 2015–2019 would persist if COVID-19 had not occurred could not be tested. Possible interactions between COVID-19 and prognosis were not accounted for, for example, excess mortality could partially explain the persistent reduction in incidence and could have led to an overestimation of expected rates. However, given that underdiagnosis is evident in a wide range of conditions and in those aged <50 years, non-presentation and recording may be the biggest issue.

### Comparison with existing literature

Observational studies conducted in Spain have reported reduced incidence of multiple chronic diseases in 2020,^20^ and substantial reductions in clinical indicators for control and treatment of chronic disease in March and April 2020.^21^ A UK-based study using primary care data reported reduced incidences of depression (47.1%) and anxiety (40.8%) in Wales, Scotland, and Northern Ireland, especially among working-age adults registered at practices in more deprived areas.^22^ The current study included longer-term data showing there is likely still a lag for most conditions as services have resumed pre-pandemic activity. Further, the pandemic has exacerbated an already high prevalence of undiagnosed COPD.^23^^,^^24^

UK pandemic guidance to postpone tests that may increase the respiratory transmission of viral infections, including spirometry, likely contributed.^25^ This might also explain the difference in lag towards the end of 2021 between asthma and COPD, as spirometry is needed to diagnose COPD whereas a diagnosis of asthma is based more on the clinical history. Reductions in hospital admissions for infectious exacerbation of COPD following the national lockdown in Wales^26^ could also in part explain the reduction in incidence rates.

The absence of deficits in recorded incidence for type 1 diabetes is likely condition-specific, rather than owing to a younger patient population, as type 1 diabetes inevitably presents soon after symptom onset and there were no indications that overall trends were confounded by age. Other studies have reported increased incidence in 2020–2021, mostly in younger patients (<18 years)^27^^–^^30^ and increased risk following COVID-19 infections^27^^,^^28^ although it is unclear if the association is causative.

### Implications for research and practice

Rectifying this backlog of case identification and consequent management deficits is likely to require specific strategic and operational planning at the level of primary care organisations. Targeted catch-up initiatives are unlikely to be feasible because of the lack of sociodemographic characterisation of the missing diagnoses. Consideration for specific resource allocation to enable healthcare staff time to be committed to searching records, testing, and screening risk groups (for example, across cardiovascular conditions) is needed. Governments and policymakers may need to identify such specific funding to tackle this workload as part of COVID-19 recovery, alongside other higher-profile patient needs such as cancer care and elective surgery.

General or condition-specific patient advocacy organisations and charitable foundations may have a role in ‘championing’ for patients with potentially relevant symptoms to present to primary care (as advocated also, for example, with potential cancer symptoms),^31^ or to seek attendance and ‘health checks’ among infrequent attenders.

Further research is ongoing to identify exactly what deficits in condition management, health outcomes, and impact on health services have occurred.

## Data

The raw data sources are described in detail in the methods, which were accessed and analysed within a Trusted Research Environment (TRE). Extracting the data from the TRE is prohibited as a condition of use. Accredited researchers can apply to access the SAIL Databank via a governed approval process and is independent of the study authors (https://saildatabank.com/). The main datasets used are: Patient Episode Dataset for Wales (PEDW) dataset, Welsh Longitudinal General Practice (WLGP) dataset, and the Welsh Demographic Service Dataset. Tabulated data and results are available for readers to access within the dashboard (link above).

## References

[1] Technical Advisory Group, Welsh Government (2021). Five harms arising from COVID-19: consideration of potential baseline measures.

[2] Sanchez-Ramirez DC, Normand K, Zhaoyun Y, Torres-Castro R (2021). Long-term impact of COVID-19: a systematic review of the literature and meta-analysis. Biomed.

[3] Carr A, Smith JA, Camaradou J, Prieto-Alhambra D (2021). Growing backlog of planned surgery due to Covid-19. BMJ.

[4] Department of Health and Social Care (2012). Long term conditions compendium of information: third edition.

[5] Watt T, Firth Z, Fisher R, Kelly E (2020). Use of primary care during the COVID-19 pandemic: patient-level data analysis of the impact of COVID-19 on primary care activity in England. The Health Foundation.

[6] Lyons RA, Jones KH, John G (2009). The SAIL databank: linking multiple health and social care datasets. BMC Med Inform Decis Mak.

[7] Ford DV, Jones KH, Verplancke JP (2009). The SAIL Databank: building a national architecture for e-health research and evaluation. BMC Health Serv Res.

[8] Jones KH, Ford DV, Thompson S, Lyons RA (2019). A profile of the SAIL Databank on the UK secure research platform. Int J Popul Data Sci.

[9] Jones KH, Ford DV, Jones C (2014). A case study of the Secure Anonymous Information Linkage (SAIL) Gateway: a privacy-protecting remote access system for health-related research and evaluation. J Biomed Inform.

[10] Rodgers SE, Demmler JC, Dsilva R, Lyons RA (2012). Protecting health data privacy while using residence-based environment and demographic data. Health Place.

[11] Rodgers SE, Lyons RA, Dsilva R (2009). Residential Anonymous Linking Fields (RALFs): a novel information infrastructure to study the interaction between the environment and individuals’ health. J Public Health (Oxf).

[12] Office for National Statistics (2022). Census geography. https://webarchive.nationalarchives.gov.uk/ukgwa/20220401215420/https:/www.ons.gov.uk/methodology/geography/ukgeographies/censusgeography.

[13] Welsh Government Statistical first release. General Medical Services contract: Quality and Outcomes Framework statistics for Wales, 2018–19. 25 September 2019. https://www.gov.wales/sites/default/files/statistics-and-research/2019-09/general-medical-services-contract-quality-and-outcomes-framework-april-2018-march-20199-599.pdf.

[14] Rafferty J, Stephens JW, Atkinson MD (2021). A retrospective epidemiological study of type 1 diabetes mellitus in Wales, UK between 2008 and 2018. Int J Popul Data Sci.

[15] Office for National Statistics Welsh Index of Multiple Deprivation (full Index update with ranks): 2019, updated 2020. https://gov.wales/welsh-index-multiple-deprivation-full-index-update-ranks-2019.

[16] Mitnitski AB, Mogilner AJ, Rockwood K (2001). Accumulation of deficits as a proxy measure of aging. Sci World J.

[17] Clegg A, Bates C, Young J (2016). Development and validation of an electronic frailty index using routine primary care electronic health record data. Age Ageing.

[18] Hollinghurst J, Fry R, Akbari A (2019). External validation of the electronic Frailty Index using the population of Wales within the Secure Anonymised Information Linkage Databank. Age Ageing.

[19] Office for National Statistics (2021). Dataset. Lower layer Super Output Area population estimates (supporting information). https://www.ons.gov.uk/peoplepopulationandcommunity/populationandmigration/populationestimates/datasets/lowersuperoutputareamidyearpopulationestimates.

[20] Sisó-Almirall A, Kostov B, Sánchez E (2022). Impact of the COVID-19 pandemic on primary health care disease incidence rates: 2017 to 2020. Ann Fam Med.

[21] Coma E, Mora N, Méndez L (2020). Primary care in the time of COVID-19: monitoring the effect of the pandemic and the lockdown measures on 34 quality of care indicators calculated for 288 primary care practices covering about 6 million people in Catalonia. BMC Fam Pract.

[22] Carr MJ, Steeg S, Webb RT (2021). Effects of the COVID-19 pandemic on primary care-recorded mental illness and self-harm episodes in the UK: a population-based cohort study. Lancet Public Health.

[23] Almagro P, Soriano JB (2017). Underdiagnosis in COPD: a battle worth fighting. Lancet Respir Med.

[24] Bastin AJ, Starling L, Ahmed R (2010). High prevalence of undiagnosed and severe chronic obstructive pulmonary disease at first hospital admission with acute exacerbation. Chron Respir Dis.

[25] Primary Care Respiratory Society (2020). PCRS Position Statement. Diagnostic work up of the patient presenting with respiratory symptoms during the COVID-19 pandemic.

[26] Alsallakh MA, Sivakumaran S, Kennedy S (2021). Impact of COVID-19 lockdown on the incidence and mortality of acute exacerbations of chronic obstructive pulmonary disease: national interrupted time series analyses for Scotland and Wales. BMC Med.

[27] McKeigue PM, McGurnaghan S, Blackbourn L (2022). Relation of incident type 1 diabetes to recent COVID-19 infection: cohort study using e-health record linkage in Scotland.. Diabetes Care.

[28] Barrett CE, Koyama AK, Alvarez P (2022). Risk for newly diagnosed diabetes >30 days after SARS-CoV-2 infection among persons aged <18 years — United States, March 1, 2020–June 28, 2021. MMWR Morb Mortal Wkly Rep.

[29] Unsworth R, Wallace S, Oliver NS (2020). New-onset type 1 diabetes in children during COVID-19: multicenter regional findings in the UK. Diabetes Care.

[30] Passanisi S, Salzano G, Aloe M (2022). Increasing trend of type 1 diabetes incidence in the pediatric population of the Calabria region in 2019‒2021. Ital J Pediatr.

[31] Smith P, Moody G, Clarke E (2022). Protocol for a feasibility study of a cancer symptom awareness campaign to support the rapid diagnostic centre referral pathway in a socioeconomically deprived area: Targeted Intensive Community-based campaign To Optimise Cancer awareness (TIC-TOC). BMJ Open.

